# Novel chia (*Salvia Hispanica* L.) residue-based substrate formulations for oyster mushroom (*Pleurotus ostreatus*) cultivation

**DOI:** 10.1007/s11274-025-04645-8

**Published:** 2025-11-15

**Authors:** Clara R. Azzam, Monica Mburu, Ahmed M. S. Hussein, Ramadan A. Arafa, Mokhtar S. Rizk, Arafat A. Abdel Latef, Mostafa M. Rady, Emad A. Salem

**Affiliations:** 1 Cell Research Department, Field Crops Research Institute, Agricultural Research Center (ARC), Giza, 12619 Egypt; 2 Centre for Food Safety and Naturopathy, Dedan Kimathi University of Technology Private Bag, 10143 Dedan Kimathi, Nyeri, 10100 Kenya; 3 Department of Food Technology, Food Industries and Nutrition Research Institute, National Research Centre, El- Buhouth Street, Dokki, Cairo, 12622 Egypt; 4 Plant Pathology Research Institute, Agricultural Research Center (ARC), Giza, 12619 Egypt; 5 Genetic Resources Department, Desert Research Center (DRC), P.O.B. 11753, Cairo, Egypt; 6 Faculty of Science, Botany and Microbiology Department, South Valley University, Qena, 83523 Egypt; 7 Botany Department, Faculty of Agriculture, Fayoum University, Fayoum, 63514 Egypt; 8 Central Laboratory for Agricultural Climate, Agricultural Research Center, Dokki, Giza, 12411 Egypt

**Keywords:** Chia residues, Edible mushroom, Mushroom yield, Nutritional quality, Functional food, JUNCAO technique

## Abstract

Chia (*Salvia hispanica*) and mushrooms represent valuable natural resources with numerous beneficial attributes as functional food ingredient with potential health benefits that support human health. The utilization of chia residues as a substrate for mushroom cultivation presents an innovative approach to supports health beneficial mushroom production. The aim of this study was, the residual biomass from chia (variety Misr 1), referred to as chia residue (CR), was evaluated as a substrate component for the cultivation of oyster mushroom (*Pleurotus ostreatus*). The CR was combined with rice straw (RS) and corn stalks (CS) through permutation and integration techniques. A total of ten deferent substrate formulations using deferent percentages of CR, RS and CS and supplemented with wheat bran, gypsum powder and calcium carbonate were prepared and evaluated using JUNCAO technology. Evaluation criteria included mushroom yield, quality, and chemical composition. Results indicated that substrates containing 86% RS or a mixture of 64% CR and 22% RS achieved complete mycelial colonization within 23 days. Primordial formation was observed by day 33 in most formulations. Formulations with balanced ratios of RS and CR (64:22 or 22:64) achieved the highest yields and improved nutritional quality, both demonstrating enhanced nutritional profiles and bioactive compound content.

## Introduction

Chia (*Salvia hispanica* L.) is a C3, summer annual alternative and innovative crop belonging to the Lamiaceae (mint) family. Native to southern Mexico, the Mediterranean Basin, and arid regions of Chile, chia naturally thrives in tropical and subtropical climates, with an optimal growth temperature range of 16–26 °C (Joseph 2014; Salman et al. 2019). Historically cultivated by indigenous peoples, chia has been reintroduced into modern agriculture due to its notable nutritional and nutraceutical properties, positioning it as a valuable functional food source (Caruso et al. 2018). In the Mediterranean Basin, chia serves as a notable example of alternative and innovative crops, alongside teff, quinoa, camelina, and nigella (Bilalis et al. 2017). It is increasingly recommended as an alternative crop for the field crop industry due to its adaptability and nutritional benefits (Ikumi et al. 2019; Kirsch et al. 2024; Azzam et al. 2025a and b). In Kenya, more than 80% of the land area is classified as arid or semi-arid, and between 2019 and 2021 about 69.5% of the population experienced moderate to severe food insecurity, with 26.9% classified as undernourished (FAO 2015; FAO 2021). Similarly, Egypt is predominantly desert, with most of its population concentrated in the Nile Valley and Delta, and is projected to face severe water scarcity driven by rapid population growth (Ahmedk et al. 2021). Given these challenges, crops such as chia, requiring 13–38% less water than alfalfa, corn, and soy (Kirsch et al. 2024), may provide nutritional and agronomic advantages.

Additionally, large-scale chia production is anticipated to generate substantial amounts of lignocellulosic residues, representing both a challenge and an opportunity. Ferdous et al. (2020) reported that the holocellulose (cellulose and hemicellulose) content in chia plant was 60.5%, confirming its high lignocellulosic potential. Since cellulose and hemicellulose are major structural components of plant cell walls and serve as the primary carbon source for fungal growth (Zhou et al. 2023; Aditya et al., 2024), chia residues can provide an effective substrate for mushroom cultivation. Alexei et al. (2022), found that chia dry stalks contain 357 g/kg cellulose, 197 g/kg hemicellulose and 72 g/kg acid deter-gent lignin.

On a global scale, lignocellulosic waste production is estimated at approximately 140 gigatons annually, much of which is incinerated or discarded, contributing to environmental degradation (Devi et al. 2023). The bioconversion of such residues through mushroom cultivation offers a sustainable waste management strategy that simultaneously provides nutritious food. Due to their low cost, renewability, and abundance, lignocellulosic residues thus represent highly suitable substrates for mushroom production.

Mushroom cultivation remains a vital economic activity worldwide, driven by rising consumer demand for nutritious and sustainable foods. The global mushroom industry has grown steadily in recent decades, driven by increasing demand for functional foods, sustainable protein alternatives, and environmentally friendly farming practices. According to FAOSTAT and USDA reports, global mushroom production surpassed 45 million tons in 2022, with China, the United States, and several European countries being the main producers (FAO 2023; USDA 2022). The Chinese JUNCAO technology, contributed in making China a leading country in mushroom production. JUNCAO technology was developed in 1983, successfully utilized wild grasses or crop residues by creating specific formulations to replace logs or sawdust from broadleaf trees for the cultivation of edible and medicinal fungi (Claude et al. 2024).

In addition to its economic value, the sector provides significant rural employment opportunities and contributes to food and nutritional security, also highlighted the important role of mushroom production in recycling agricultural wastes into high-value food products, further reinforcing its relevance to sustainable agriculture (Royse et al. 2017). Edible mushrooms are rich sources of crude soluble fiber, proteins, vitamins, and minerals, including calcium, magnesium, potassium, zinc, iron, and selenium, while being low in starch and caloric content, thus conferring numerous health benefits (Naim et al. 2020a; Atila et al. 2024). For rural populations reliant on staple crops that are often deficient in micronutrients like iron and zinc, mushrooms can serve as a dietary intervention to combat micronutrient malnutrition, especially among pregnant women and children (Mostafa et al. 2023). These factors collectively underscore the considerable economic significance of mushroom farming within the global market.

The genus *Pleurotus* includes economically significant species that exhibit a relatively short cultivation cycle compared to other commercially cultivated fungi, and are widely distributed across temperate, subtropical, and tropical regions (Širic et al. 2022). *Pleurotus* spp. are valued for their high nutritional content, including dietary fiber, proteins, amino acids, vitamins, and minerals (Naim et al. 2020b). Moreover, they are recognized for their medicinal properties, attributed to bioactive compounds such as polysaccharides, which exhibit a range of biological activities including antibacterial, antiviral, hematological, hypocholesterolemic, antitumor, immunomodulatory, and blood glucose regulatory effects (Abou Fayssal et al. 2021; Werghemmi et al. 2022; Abou Fayssal and Yordanova 2024). Furthermore, it demonstrates versatility in substrate selection, as it can utilize a broad spectrum of lignocellulosic materials, often considered agricultural or industrial waste, such as straw, sawdust, husks (Ritota and Manzi 2019). Consequently, cultivated oyster mushrooms facilitate the production of high-quality protein sources while simultaneously valorizing agricultural by-products. This dual functionality positions mushrooms as a valuable nutritional resource, particularly in the context of sustainability imperatives and the increasing global population.

The study was performed in Central Laboratory of Agricultural Climate, Egypt and aimed to evaluate the potential of chia plant residues, comprising the entire plant biomass remaining after seed harvesting, as a substrate for the cultivation of *Pleurotus ostreatus*. It intends to develop multiple substrate formulations to assess their effects on mycelial growth, mushroom yield, and morphological features of the fruiting bodies, with comparisons made to conventional substrates such as corn stalks and rice straw, which will serve as control treatments. Additionally, the study aimed to analyze the proximate composition, mineral content, and functional properties of the harvested mushroom fruiting bodies so as to determine their nutritional and bioactive characteristics.

## Materials and methods

### Preparation of substrate formulation

This study was a part of the international research project titled “Integrated Chia and Oyster Mushroom System for Sustainable Food Value Chain for Africa” (CHIAM), in which different countries including Kenya (the project coordinator) and Egypt are participating. Furthermore, also was conducted at the Central Laboratory for Agricultural Climate (CLAC), Agricultural Research Center (ARC), Ministry of Agriculture, Dokki, Egypt, within a controlled mushroom cultivation greenhouse from January to March 2024. The objective was to assess the productivity and quality attributes of an oyster mushroom strain from the CLAC Mushroom Fungi Unit collection (*Pleurotus ostreatus* var. florida (Jacq: Fr) Kummer 14).

Spawn was prepared using the sorghum grain method following the JUNCAO technique (Zhanxi and Dongmei 2008; Claude et al. 2024). The substrate components comprised chia residues (Misr 1 variety, the first registered chia variety in Egypt by ARC, obtained from field experiments of the CHIAM project after harvesting), rice straw, and corn stalks (sourced from Kafr El Sheikh Governorate by CLAC). These materials formulated ten different substrate formulations (SFs). Additives, including wheat bran, gypsum, and calcium carbonate, were incorporated at fixed proportions of 10%, 2%, and 2%, respectively (Zhanxi and Dongmei 2008; Claude et al. 2024). The remaining 86% of the substrate was allocated either to a single component or divided between two components in proportions of 25% and 75%, which corresponded to 22% and 64% of the total mixture when expressed as percentages of the entire substrate. Formula 1 functioned as the control treatment, whereas Formulas 4 and 5 represented conventional cultivation practices commonly used worldwide, particularly in China, the origin of JUNCAO technology (Zhanxi and Dongmei 2008; Claude et al. 2024). The remaining formulations incorporated different proportions of chia residues, as detailed in Table 1.

**Table 1:** Substrate formulations (SFs) and their component percentages (%) evaluated in this study

SF	RS (%)	CS (%)	CR (%)	WB (%)	GP (%)	CC (%)
SF 1	100	-	-	-	-	-
SF 2	43	-	43	10	2	2
SF 3	-	43	43	10	2	2
SF 4	-	86	-	10	2	2
SF 5	86	-	-	10	2	2
SF 6	-	-	86	10	2	2
SF 7	64	-	22	10	2	2
SF 8	22	-	64	10	2	2
SF 9	-	64	22	10	2	2
SF 10	-	22	64	10	2	2

*SF* Substrate formulations, *RS* Rice straw, *CS* Corn Stalks, *CR* Chia residues, *WB* Wheat Bran, *GP* Gypsum Powder and *CC* Calcium Carbonate

### Mushrooms cultivation process

**Fig. 1:**
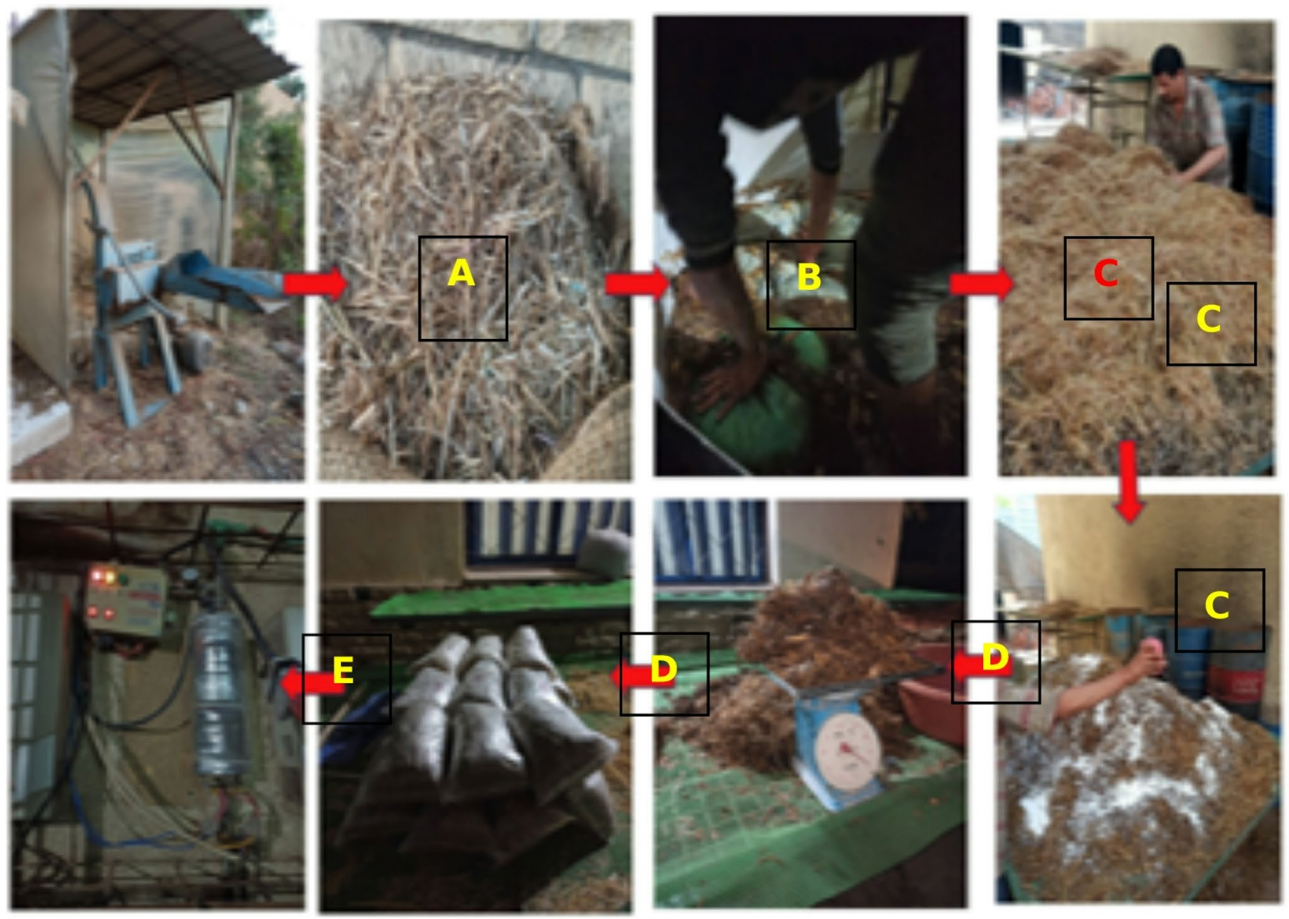
Sequential steps involved in the preparation of the substrate formulations. (**A**) Chopping of rice straw, corn stalks, and chia residues into small pieces; (**B**) Washing and soaking the chopped materials in tap water to adjust moisture content; (**C**) Mixing of substrate components with additives (wheat bran, gypsum, and calcium carbonate) to form homogeneous formulations; (**D**) Packing of 1,700 g moist substrate into polypropylene bags (25 × 15 cm) and sealing; (**E**) Pasteurization of bags using steam at 110 °C for 3 h before inoculation with mushroom spawn

The sequential procedure for preparing the substrate formulations is illustrated in Fig. 1. The substrate components: rice straw (RS), corn stalks (CS), and chia residues (CR) were initially chopped (Fig. 1A) and rinsed by soaking in tap water (Fig. 1B). Moisture content was adjusted to achieve the desired consistency were left to drain until 1–3 drops were released when squeezing by hand. Ten substrate formulations were prepared as shown in Table 1 and thoroughly homogenized (Fig. 1C). The moisture content across treatments ranged from 58% to 68%, while pH values varied between 6.8 and 7.2. Substrates were packed into polypropylene bags measuring 25 cm in length and 15 cm in diameter, each containing 1,700 g of moist substrate (Fig. 1D). For each formulation, nine bags were prepared, arranged as three replicates with three bags per replicate. The bag openings were sealed and placed on shelves inside a steam chamber with a capacity of 1.2 m³. Substrates were subjected to moist-heat sterilization using a steam generator (Universal Way Company) operating at an outlet temperature of 110 °C for 3 h. The temperature inside representative bags was monitored to ensure that the target level was achieved and maintained throughout the treatment. After completion, the bags were allowed to cool gradually to ambient temperature within the closed chamber to minimize the risk of recontamination.

Mushroom spawn of *Pleurotus ostreatus* was prepared on sterilized sorghum grains following standard procedures. Cleaned grains were boiled until partially softened, drained, and supplemented with 2% gypsum and 1% calcium carbonate (w/w) to prevent clumping and stabilize pH. The grains were filled into 500 g polypropylene bags, sterilized at 121 °C for 2 h, and aseptically inoculated with actively growing mycelial plugs obtained from pure *P. ostreatus* cultures. Bags were incubated at 25 ± 2 °C in darkness for 12–15 days until complete colonization with white, healthy mycelium. Following cooling of the cultivation substrates to ambient temperature, approximately 50 g of this spawn was aseptically inoculated onto the upper 5 cm layer of each bagged substrate, and the openings were securely tied to allow gas exchange while minimizing contamination risk.

**Fig. 2:**
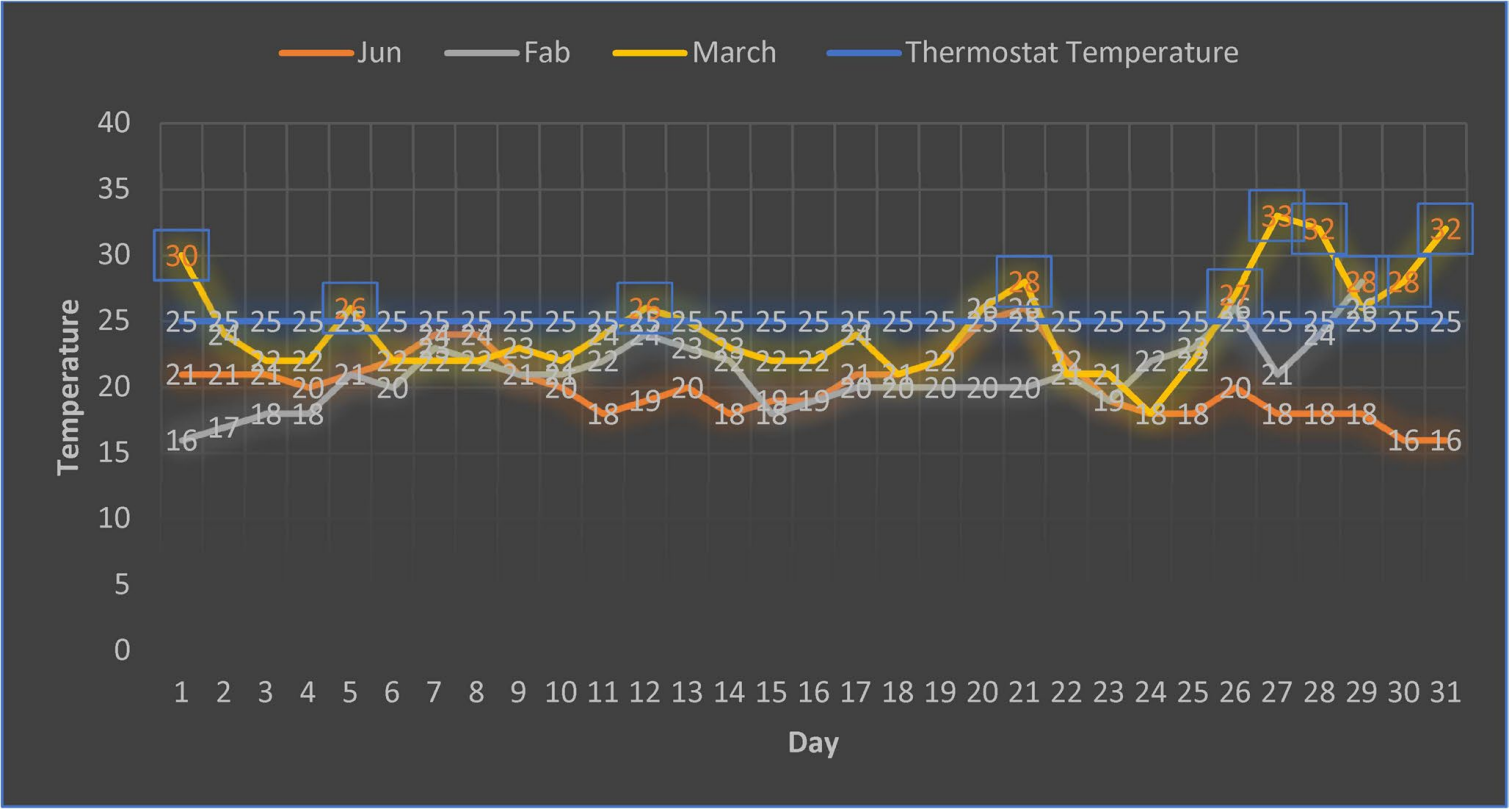
Temperature profile within the mushroom cultivation greenhouse throughout the production period (January to March2024). *Temperatures in inside a blue box mean automatic operation of the cooling system inside mushroom cultivation greenhouse to reduce it to the thermostat temperature (25 ˚C)

The remaining formulations included varying proportions of chia residues (CR), as detailed in Table 1, to systematically evaluate their potential to replace conventional substrates while maintaining optimal physical and nutritional characteristics for *Pleurotus ostreatus* growth. Following incubation, the inoculated bags were transferred to a controlled mushroom cultivation greenhouse for the fruiting phase. During this stage, humidity was maintained at 90% using a hygrostat, and fluorescent lighting provided illumination. Temperature regulation was achieved via a thermostat, ensuring that it did not exceed 25 °C (see Fig. 2). An evaporative pad cooling system (Mussa et al. 2024) was employed to maintain optimal temperature conditions and to facilitate the initiation and development of fruiting bodies.

The bags were grouped into three sets of three bags each, serving as replicates, and were randomly distributed on horizontal shelves. Incubation was conducted in darkness at around 27 °C, a temperature favorable for mycelial colonization, until complete substrate colonization was achieved.

Upon completion of colonization, environmental parameters were adjusted to induce fruiting. The temperature was lowered to 23 °C, the relative humidity was maintained at 90%, and the lighting was activated. Light intensity, measured with a Luxmeter (Lutron LX 105), ranged from 163 to 208 lx. To facilitate pinhead formation and fruiting body development, five openings were created on the upper surface of each bag using a cutter.

The mycelial growth rate was determined at two and three weeks post-inoculation by tracing the radial expansion of the colony inside each bag using a ruler. Measurements were taken from four equidistant sides (top, bottom, left, and right), and the mean value was calculated to ensure reproducibility under controlled incubation temperatures of 25–27 °C.; The number of days elapsed from inoculation until the substrate within the bags was fully colonized by mycelium; Primordial initiation, which refers to the number of days from inoculation until the emergence of pinheads, indicating the onset of fruit body development, under environmental conditions of 23–24℃ (Zhanxi and Dongmei 2008; Claude et al. 2024; Mesele et al. 2024).

### Yield, and morphological characteristics of fruit bodies

To evaluate mushroom productivity, the yield was measured by recording the fresh weight of mushrooms harvested from each bag, and the total yield per formulation was calculated by summing the weights across flushes. Meanwhile, the biological efficiency (BE) was assessed as the ratio of the fresh weight of mushrooms to the dry weight of the substrate, expressed as a percentage (BE %). In addition, morphological traits were characterized by measuring pileus diameter (cm), stipe length (cm), and stipe diameter (cm) using a digital caliper for accuracy (Zhanxi and Dongmei 2008; Claude et al. 2024; Mesele et al. 2024).

### Chemical analysis of the dried fruit bodies

The harvested fruiting bodies from each treatment were dried at 50 °C in a Heraeus UT6120 drying oven for 48 h. Following drying, samples were ground using a grain mill (Bioblock Techno-Scientific M20). The resulting powder was subjected to the following analyses.

#### Proximate compositions

Protein, fat, ash, and crude fiber contents were determined according to (AOAC, 2005) standards. Carbohydrate content was calculated by subtracting the sum of protein, fat, ash, and crude fiber percentages from 100%.

#### Mineral content

Sodium (Na), calcium (Ca), and potassium (K) levels were measured in ash solution samples using an emission flame photometer, following the methodology outlined by (Brown and Lillel 1946). Phosphorus (P) was estimated as described by (Chapman and Pratt 1962). Iron (Fe) and zinc (Zn) concentrations, expressed as mg per 100 g of sample, were determined spectrophotometrically using an atomic absorption spectrophotometer (Model Spectronic 21 D), according to (Jackson 1973).

#### Phytochemical and antioxidant analyses

For phenolics, flavonoids, and antioxidant activity, extracts were prepared with methanol. One milliliter of sample was mixed with 100 ml of methanol and homogenized using an Ultra-Turrax homogenizer. The homogenates were stored at 4 °C for 12 h, and then centrifuged at 10,000 rpm for 20 min. Supernatants were collected and stored at −20 °C until analysis.

#### Total phenolic content

Total phenolic content was determined via the Folin-Ciocalteu method (Singleton et al. 1999). Briefly, 500 µl of extract was mixed with 250 µl of Folin-Ciocalteu reagent, incubated for 5 min, and then neutralized with 1.25 ml of 20% sodium carbonate solution. After 40 min of incubation, absorbance was measured at 725 nm against a blank. Phenolic content was quantified using a gallic acid calibration curve and expressed as milligrams of gallic acid equivalents (GAE) per gram of sample.

#### Total flavonoid content

Total flavonoid content was assessed following Zhuang et al. (1992). A 250 µl aliquot of 5% NaNO₂ was combined with 500 µl of extract. After 6 min, 2.5 ml of 10% AlCl₃ solution was added, followed by 1.25 ml of 1 M NaOH after 7 min. The mixture was centrifuged at 5000 g for 10 min, and the supernatant’s absorbance was measured at 510 nm against a blank. Total flavonoid content was expressed as milligrams of catechin equivalents (CAE) per gram of sample.

#### Antioxidant activity using DPPH radical scavenging assay

The antioxidant activity of the methanolic extract of mushroom powder was determined in terms of hydrogen donating or radical scavenging ability using the stable radical DPPH (2, 2-diphenyl-1-picrylhydrazyl) carried out according to the method described by Menaga et al. (2013). Radical scavenging activity was calculated using the formula: DPPH scavenging rate (%) = (Control OD – Sample OD/Control OD) × 100. The DPPH solution was prepared at 0.1 mM concentration in methanol, and mushroom extracts were tested at a final concentration of 1 mg/ml.

### Statistical analysis

Statistical analysis of the growth and fructification measurements was performed using one-way analysis of variance (ANOVA) with CoStat for Windows (version 6.311). The significance of differences between treatments, such as yield, biological efficiency, and morphological traits, was determined using the least significant difference (LSD) test, which was applied only after obtaining significant F-values from ANOVA, as it provides higher sensitivity in detecting treatment effects in small-scale factorial experiments. *P* values less than 0.05 were considered statistically significant (McClave and Benson 1991). Standard deviations (SD) were calculated using Microsoft Excel to represent variability among replicates.

## Results

### Mycelial growth and primordial initiation

Data presented in Table 2 show significant variation in mycelial growth among different substrate formulations (SFs). ANOVA confirmed these differences (Week 2: F = 45.3, df = 9, 20, *P* < 0.001; Week 3: F = 38.7, df = 9, 20, *P* < 0.001). After two weeks, SF 8 exhibited the fastest growth (20.6 ± 0.5 cm, a), followed by SF 6 and SF 7 (17.5 ± 0.4 cm, b), whereas SF 1 and SF 3 were the slowest (7.8 ± 0.3 and 7.1 ± 0.2 cm, f). By Week 3, SF 5, SF 8, and SF 10 reached the highest growth (23.1–24.3 ± 0.4–0.6 cm, a), exceeding SF 3 (16.0 ± 0.3 cm, d) by 7.1–8.3 cm (*P* < 0.05), while SF 4 and SF 7 showed intermediate growth (20.8–21.5 ± 0.3–0.4 cm, ab–b). Values are mean ± SD (*n* = 3 bags × 3 replicates), and letters indicate significant differences according to LSD at *P* < 0.05.

**Table 2:** Mycelial growth progress (cm) during the incubation period (with bag length of 25 cm), number of days for complete colonization of different substrate formulation and primordial initiation

Substrate formulations	Mycelial growth progress (cm)			Number of days for complete colonization	Primordial initiation (Days)
	Week 2		Week 3		
SF 1		7.80 ± 0.3^f^	16.2 ± 0.3^d^	26 ± 0.5^e^	39 ± 0.6^d^
SF 2		13.8 ± 0.4^d^	20.8 ± 0.3^b^	25 ± 0.4^cd^	39 ± 0.5^d^
SF 3		7.1 ± 0.2^f^	16.0 ± 0.3^d^	28 ± 0.5^f^	43 ± 0.6^e^
SF 4		15.3 ± 0.3^c^	20.8 ± 0.4^ab^	26 ± 0.4^de^	33 ± 0.5^b^
SF 5		10.4 ± 0.4^e^	24.0 ± 0.5^a^	23 ± 0.3^a^	33 ± 0.4^b^
SF 6		17.5 ± 0.4^b^	21.7 ± 0.4^b^	25 ± 0.4^cd^	33 ± 0.4^b^
SF 7		17.5 ± 0.4^b^	21.5 ± 0.3^ab^	25 ± 0.4^cd^	33 ± 0.4^b^
SF 8		20.6 ± 0.5^a^	24.3 ± 0.6^a^	23 ± 0.3^a^	33 ± 0.4^b^
SF 9		11.4 ± 0.3^d^	18.5 ± 0.3^c^	28 ± 0.5^f^	43 ± 0.6^e^
SF 10		14.8 ± 0.4^bc^	23.1 ± 0.4^a^	24 ± 0.4^b^	33 ± 0.4^b^

Mean values within a column followed by the same letters are considered not significantly different at *P* values < 0.05 by using the LSD test

Complete colonization of the substrate bags was first achieved by SF 5 and SF 8 after 23 days, followed by SF 10 after 24 days, whereas SF 3 and SF 9 required 28 days. Primordial initiation, indicated by pinhead formation, generally occurred within 33 days for most SFs, but SF 1, SF 2, SF 3, and SF 9 showed delayed initiation at 39–43 days. These results indicate that substrate formulation strongly affects both the rate of mycelial growth and the timing of primordial formation.

### Fructification characteristics

Table 3 presents yield parameters of the produced fruit bodies. In terms of fresh weight (FW) of fruit bodies per bag, the treatment SF 6 exhibited the significantly highest FW (137.57 ± 7.65, *P* < 0.05), nevertheless having a comparatively lower percentage of dry weight (DW %), (8.99 ± 0.51, *P* > 0.05). Following SF6, the treatment SF4 also recorded a significant fresh weight per bag; however, it gave a lower dry weight% (DW %), (9.39 ± 0.50, *P* > 0.05). Conversely, treatments SF7 and SF8 demonstrated the highest DW% (11.10 ± 0.75 and 11.12 ± 0.79, respectively, *P* < 0.05), despite it being a low value for FW. Treatment SF 1 showed the lowest values for both FW per bag and DW %. In terms of biological efficiency (BE), treatment SF 8 achieved the highest performance, closely followed by SF 7 (43.27 ± 4.3 and 36.63 ± 4.4, respectively, *P* < 0.05). Conversely, SF 1 exhibited the lowest BE among all treatments. Regarding the number of clusters per bag, SF 6 produced the most favorable results, with subsequent high yields observed in SF 4, SF 5, and SF 7. The parameter measuring the number of pilei per cluster revealed that SF 3 had the highest count, although it also recorded the smallest average pileus width. In contrast, SF 8 produced fewer pilei per cluster (4.88 ± 3.85, *P* > 0.05) but with the largest pileus diameter (10.25 ± 2.16, *P* < 0.05). The treatments SF 6, yielded the highest results concerning the number of clusters per bag (3.33a ± 0.45, *P* < 0.05), followed by SF 4, SF 5, and SF 7. Although SF 1 and 2 showed low values in the previous parameters, they exhibited shorter stipe lengths (2.45 ± 0.76, 2.69 ± 0.82, *P* > 0.05, respectively). In contrast, SF 10 demonstrated the highest stipe length (4.62 ± 1.13). Furthermore, SF 4 exhibited the smallest stipe diameter, while SF 1 and 9 showed the largest stipe diameters. Treatment SF 3 displayed low significance in DW %, but showed notable results in stipe dimensions.

**Table 3:** Yield parameters for *Pleurotus ostreatus* var Florida cultivated on different substrate formulation

SFs	FW/ Bag (g)	DW/Bag (g)	DW %	BE (%)	No. of clusters/Bag	No. of Pileus/Cluster	Pileus width (cm)	Stipe length	Stipe diameter
SF 1	67.99 ± 19.09^f^	6.28 ± 2.07^f^	9.23 ± 1.58^cd^	23.57 ± 3.2^f^	1.50 ± 0.53^d^	6.25 ± 2.16^d^	8.31 ± 3.62^3bc^	2.45 ± 0.76^d^	1.56 ± 0.53^a^
SF 2	82.22 ± 25.79^e^	7.65 ± 2.74^e^	9.30 ± 1.01^cd^	27.13 ± 2.3^e^	1.50 ± 1.34^d^	8.54 ± 4.48^b^	8.05 ± 3.22^bc^	2.69 ± 0.82^cd^	1.30 ± 0.55^b^
SF 3	113.56 ± 26.20^b^	11.72 ± 2.40^ab^	10.32 ± 0.36^b^	23.63 ± 3.5^f^	1.28 ± 0.49^d^	10.64 ± 3.79^a^	7.17 ± 1.49^c^	4.14 ± 1.78^ab^	1.48 ± 1.38^ab^
SF 4	120.39 ± 18.80^ab^	11.30 ± 2.00^ab^	9.39 ± 0.50^cd^	27.13 ± 3.7^e^	2.70^ab^ ± 1.4	5.73 ± 2.6^de^	7.35 ± 4.0^c^	3.34 ± 1.20^bcd^	1.04 ± 0.3^d^
SF 5	77.63 ± 19.31^e^	7.95 ± 2.41^e^	10.82 ± 2.15^ab^	30.23 ± 5.2^c^	2.36 ± 0.51^b^	3.45 ± 1.17^f^	7.42 ± 1.63^c^	3.67 ± 1.60^abc^	1.12 ± 0.34^c^
SF 6	137.57 ± 7.65^a^	12.37^a^ ± 1.15	8.99 ± 0.51^de^	28.60 ± 2.2^d^	3.33 ± 0.45^a^	4.58 ± 0.38^ef^	4.70 ± 2.13^d^	3.84 ± 1.03^ab^	1.12 ± 0.52^c^
SF 7	98.74 ± 35.44^c^	10.52 ± 3.29^bc^	11.10 ± 0.75^a^	36.63 ± 4.4^b^	2.21 ± 1.03^bc^	7.62 ± 6.05^bc^	5.84 ± 2.39^d^	3.19 ± 1.04^bcd^	0.99 ± 0.45^f^
SF 8	85.15 ± 15.42^cd^	9.32 ± 1.70^cd^	11.12 ± 0.79^a^	43.27 ± 4.3^a^	1.58 ± 0.78^cd^	4.88 ± 3.85^e^	10.25 ± 2.16^a^	3.87 ± 1.32^ab^	1.22 ± 0.48^b^
SF 9	96.13 ± 30.40^c^	10.56 ± 3.30^b^	9.36 ± 0.80^cd^	27.97 ± 3.3^d^	1.61 ± 0.80^cd^	6.27 ± 6.40^d^	9.95 ± 2.8^a^	3.34 ± 1.3^bcd^	1.50 ± 0.40^a^
SF 10	92.20 ± 25.73^cd^	7.27 ± 2.22^ef^	10.50 ± 0.66^b^	23.67 ± 2.1^f^	1.50 ± 0.74^d^	6.39 ± 3.93^cd^	9.34 ± 2.69^ab^	4.62 ± 1.13^a^	1.46 ± 0.54^ab^

*FW and DW* fresh and dry weight, respectively, *BE* Biological Efficiency, *SF* Substrate formulations

Mean values within a column followed by the same letters are considered not significantly different at *P* values < 0.05 by using the LSD test

### Chemical analysis of mushroom powder

The proximate composition of the mushroom powder samples is summarized in Tables 4 and 5. Table 4 presents the contents of ash, protein, fat, fiber, carbohydrates, and energy. The total ash content across samples ranged from 6.96 to 9.65%, with SF 8 exhibiting the highest ash percentage (9.65%), while SF 4 and SF 1 showed the lowest values (6.96% and 7.99%, respectively).

**Table 4:** Proximate compositions, total phenols, total flavonoids, phytochemical and antioxidant activity mean values of the mushroom powder samples

Substrate formulations	Ash	Protein	Fat	Fiber	Carbohydrate	Energy Kcal/100 gm	Total Phenols (mg GAE/g)	Total flavonoid (mg CAE/gm)	Antioxidant activity (DPPH) (%)
**SF 1**	7.99±0.19^d^	26.90±0.15^c^	1.78±0.10^e^	8.33±0.11^c^	55.00±0.45^a^	262.92±3.84^c^	3.02±0.15^b^	5.19±0.05^d^	42.15±0.12^f^
**SF 2**	9.24±0.22^a^	27.50±0.18^b^	1.65±0.06^f^	8.65±0.09^b^	52.96±0.48^b^	254.19±3.68^c^	3.13±0.13^b^	5.65±0.07^d^	44.11±0.15^d^
**SF 3**	9.61±0.16^a^	28.10±0.22^b^	1.85±0.09^e^	9.05±0.13^b^	51.39±0.16^c^	250.31±4.75^c^	3.32±0.11^a^	6.05±0.10^c^	43.19±0.10^e^
**SF 4**	6.96±0.09^e^	26.15±0.22^d^	2.60±0.01^c^	9.17±0.19^ab^	55.12±0.26^a^	348.48±2.87^a^	3.27±0.19^a^	6.35±0.15^c^	45.35±0.21^c^
**SF 5**	8.77±0.17^b^	24.50±0.258^e^	2.15±0.03^d^	8.75±0.08^b^	55.83±0.58^a^	340.67±4.21^b^	3.36±0.22^a^	6.65±0.09^b^	46.10±0.17^b^
**SF 6**	9.00±0.10^b^	29.18±0.25^a^	2.35±0.06^d^	9.35±0.10^a^	50.12±0.39^c^	338.35±3.65b	3.51±0.10^a^	7.15±0.11^b^	47.28±0.19^a^
**SF 7**	8.58±0.13^c^	27.35±0.29^c^	2.75±0.03^b^	9.15±0.29^b^	52.17±0.42^b^	342.83±4.45^a^	3.37±0.25^a^	6.90±0.19^b^	45.80±0.29^bc^
**SF 8**	9.65±0.07^a^	29.75±0.42^a^	2.88±0.05^b^	9.50±0.37^a^	50.47±0.55^c^	341.8. ±5.96^b^	3.41±0.32^a^	7.95±0.30^a^	46.65±0.34^b^
**SF 9**	9.18±0.13^b^	28.10±0.27^b^	2.70±0.07^c^	9.65±0.13^a^	50.37±0.60^c^	338.18±5.44^b^	3.62±0.17^a^	7.45±0.17^a^	48.25±0.32^a^
**SF 10**	9.08±0.15^b^	28.35±0.35^b^	3.05±0.02^a^	9.82±0.33^a^	53.30±0.65^b^	359.65±5.88^a^	3.48±0.41^a^	7.55±0.38^a^	46.92±0.39^b^

**Table 5:** Mineral contents (mg/100 g dry Matter) of dried mushroom samples

Substrate formulations	Macro-elements					Micro-elements		
	Ca	K	Mg	*P*	Na	Fe	Zn	Se
SF 1	30.12 ± 0.15^b^	1105 ± 3.15^c^	135.10 ± 0.65^b^	680.00 ± 1.25^f^	122.15 ± 1.22^b^	48.19 ± 0.19^c^	22.15 ± 0.10^b^	7.15 ± 0.10^c^
SF 2	33.10 ± 0.17^a^	1095 ± 2.17^c^	139.15 ± 1.15^a^	695.00 ± 1.19^e^	128.10 ± 1.65^b^	47.12 ± 0.29^c^	23.15 ± 0.18^b^	7.22 ± 0.25^c^
SF 3	35.11 ± 0.12^a^	1122 ± 1.95^b^	138.25 ± 0.17^a^	710.00 ± 2.10^d^	130.11 ± 1.15^a^	50.19 ± 0.25^b^	24.14 ± 0.17^a^	7.80 ± 0.13^b^
SF 4	28.14 ± 0.19^c^	1120 ± 2.65^b^	135.11 ± 1.65^a^	750.50 ± 1.01^b^	125.70 ± 1.15^b^	52.15 ± 0.22^b^	24.16 ± 0.32^a^	8.50 ± 0.12^b^
SF 5	31.35 ± 0.10^b^	1130 ± 3.28^a^	128.20 ± 1.17^b^	740.00 ± 1.22^c^	120.00 ± 0.95^c^	54.10 ± 0.19^a^	25.20 ± 0.16^a^	8.20 ± 0.16^b^
SF 6	32.25 ± 0.16^b^	1115 ± 4.20^b^	140.29 ± 1.19^a^	735.00 ± 1.20^c^	125.20 ± 1.05^b^	52.15 ± 0.17^b^	24.38 ± 0.14^a^	8.70 ± 0.15^a^
SF 7	28.50 ± 0.25^c^	1125 ± 2.90^b^	138.28 ± 1.16^a^	755.00a ± 1.22^b^	127.32 ± 1.09^b^	53.19 ± 0.29^b^	24.92 ± 0.44^a^	8.90 ± 0.15^a^
SF 8	28.90 ± 0.17^c^	1130 ± 3.15^a^	140.18 ± 1.72^a^	760.00 ± 1.18^a^	130.25 ± 1.35^a^	55.31 ± 0.39^a^	25.22 ± 0.52^a^	9.05 ± 0.19^a^
SF 9	30.15 ± 0.15^b^	1135 ± 3.50^a^	142.35 ± 1.90^a^	760.00 ± 1.30^a^	129.27 ± 1.65^a^	56.20 ± 0.22^a^	25.60 ± 0.11^a^	8.80 ± 0.22^a^
SF 10	29.25 ± 0.13^c^	1140 ± 3.60^a^	143.26 ± 2.05^a^	765.22 ± 1.32^a^	135.13 ± 1.42^a^	57.56 ± 0.52^a^	25.75 ± 0.69^a^	9.42 ± 0.18^a^

Protein content varied between 24.15 and 29.75%. The highest protein concentration was observed in the fruit bodies from SF 8 (29.75%), which aligns with the highest productivity and quality metrics. This was followed by SF 6 (29.18%). These results suggest that the inclusion of chia residues enhanced protein content, particularly in treatments containing 64 and 86% chia residues, respectively. The combination of chia residues with rice straw yielded better protein levels than chia residues alone. The lowest protein content was found in SF 5, which contained 86% rice straw. This increase may be attributed to the higher availability of nitrogenous compounds in chia residues compared with conventional substrates, which could support greater protein synthesis in mushroom mycelia.

Fat content in the samples ranged from 1.78 to 3.05%, with SF 10 exhibiting the highest fat level (3.05%). Other treatments containing chia residues, such as SF 8, 7, and 9, showed moderate fat levels, whereas SF 1, 2, and 3 had the lowest fat contents.

Crude fiber, an important dietary component, did not differ significantly across most treatments (NS, *P* > 0.05), ranging from 9.05 to 9.82%. However, treatments SF1, SF2, and SF5 displayed comparatively lower fiber values (*P* < 0.05). The total carbohydrate content varied significantly from 50.12% in SF6 to 55.83% in SF5 (*P* < 0.05).

Based on these findings, SF 8 demonstrated the most favorable nutritional profile, particularly in terms of protein content. Energy values were estimated considering the contents of crude protein, carbohydrates (each providing 4 kcal per gram), and fat (providing 9 kcal per gram). The energy content across samples ranged from 250.31 to 359.65 kcal per 100 g.

### Total phenolic Content, Flavonoids, and antioxidant activity of mushroom powder

The bioactive compound analysis of the dried mushroom samples is summarized in Table 4. The total phenolic content was quantified in gallic acid equivalents (mg GAE/g), while the total flavonoids were expressed as catechin equivalents (mg CAE/g). The samples exhibited total phenolic contents ranging from 3.02 to 3.62 mg GAE/g dry weight. Notably, treatments SF 1 and SF 2 displayed the lowest phenolic levels, which correlates with their inferior vegetative growth parameters. Conversely, SF 9 showed the highest phenolic content, followed by SF 8 and SF 10, both of which demonstrated favorable vegetative performance.

The total flavonoid content in the mushroom methanolic extracts ranged from 5.19 to 7.95 mg CAE/g, with the highest flavonoid concentrations observed in SF 8, SF 9, and SF 10.

Regarding antioxidant activity, measured via 2, 2-diphenyl-1-picrylhydrazyl (DPPH) radical scavenging assay, inhibition percentages ranged from 42.15 to 48.25%. The greatest antioxidant activity was recorded in SF 9, closely followed by SF 8 and SF 10.

### Mineral content of mushroom powder

The concentrations of macro- and micronutrients in the studied edible mushroom samples are detailed in Table 5. Notably, treatments with superior vegetative growth parameters, such as SF 8, SF 9, and SF 10, also exhibited elevated levels of essential minerals, including potassium (K), magnesium (Mg), phosphorus (P), iron (Fe) and zinc (Zn). Additionally, treatments SF 3, SF 8, and SF 10 showed the highest sodium (Na) concentrations, while the maximum selenium (Se) content was detected in SF 8 and SF 10.

The calcium (Ca) content across the samples ranged from 28.14 to 35.11 mg/100 g, with the highest levels observed in treatments exhibiting lower vegetative parameters, specifically SF 3, followed by SF 2 and SF 1. Magnesium concentrations varied from 128.20 to 143.26 mg/100 g, and potassium levels ranged between 1095 and 1140 mg/100 g. Sodium content fluctuated between 122.15 and 135.13 mg/100 g.

## Discussion

### Mycelial growth and primordial initiation

To our knowledge, previous studies have mainly evaluated chia seed or mucilage as substrate additives (Colmenares-Cruz et al. 2017; Faraj and Nouri 2024). However, no peer-reviewed reports were found on the use of whole-plant chia residues (stalks, leaves, and post-harvest seed remnants) as primary substrates for *Pleurotus* spp. Comparable research has focused on other agro-residues, such as wheat straw, maize stalks, or soybean residues, which were optimized for mushroom production (Wu et al. 2019; Zeng et al. 2023). This highlights the potential novelty of the current study.

As well-known, mushrooms are affected in its growth by many factors, basically, environmental factors, type of substrate and supplementation type and rate (Nguyen and Ranamukhaarachchi 2019). *Pleurotus* sp. exhibits rapid growth across a broad temperature range (10–30 ° C) and thrives at pH level of 6.0–8.0 (Aditya et al. 2024), therefore, exploring effect of studied substrates on mycelial growth and primordial initiation appearing necessary. Data of Table 2 refers to that SF 5 (86% RS) and SF 8 (64% CR and 22% RS) are represented suitable substrate in which the mycelium growing of used strain of *Pleurotus* sp. is significantly faster than other substrates (23 days), however, SF 8 gave the highest percentage of dry weight and significant results in other parameters which give it the preference over SF 5 which is used as tradition substrate. Primordial initiation, indicated by pinhead formation, is influenced by environmental factors (Hakizimana et al. 2022). In the present study, greenhouse conditions such as relative humidity, light, and temperature were controlled; therefore, the observed variation in primordia formation among SFs is more likely attributable to substrate composition. Factors such as moisture retention capacity, pH buffering from CaCO₃ and gypsum, and substrate porosity could have driven these differences. This interpretation is in line with Chukwu et al. (2022), who found that adding different percentages of lime to substrates significantly affected the time required for primordial initiation in *Pleurotus* spp. Our results showed that primordial initiation generally occurred within 33 days across most treatments. However, SF1, SF2, SF3, and SF9 exhibited delayed primordial initiation, occurring after 39, 39, 43, and 43 days, respectively. This delay can be attributed to the type of substrate and the supplementation level. Treatments with lower proportions of chia residues (22%) or higher corn stalk content tended to have reduced moisture retention and slower nutrient release, which prolonged primordia formation. In addition, differences in supplementation with CaCO₃ and gypsum may have altered pH buffering and substrate porosity, further influencing the initiation period. These observations suggest that both substrate composition and supplementation rate interact to regulate the timing of primordia formation.

These variations in growth and primordium initiation are likely related to the nutritional composition and physicochemical properties of the substrates. Chia residues, being rich in cellulose, hemicellulose, proteins, and minerals, provided more accessible carbon and nitrogen sources and a higher water-holding capacity, which supported faster colonization and earlier primordium formation (as observed in SF8 and SF7). By contrast, substrates with lower nutrient density or higher lignin content delayed colonization, since lignin is less readily degraded by fungal enzymes. These factors collectively explain the differences observed among formulas.

### Fructification characteristics

Results on Table 3 revealed that SF 4 (86% CS) and SF 6 (containing 86% CR) induce absorbing more humidity at the expense of storage of dry matter, where it give high FW/bag, while, in the same time gave low level of % of D.W (*P* > 0.05) and insignificancy in the other growth parameters. This may due to the high water holding capacity of the substrates CS and CR. (Ritota and Manzi 2019) mentioned that high moisture-holding capacity of some agricultural by-products lowing yields of mushrooms, while low water holding capacity, such as in wheat straw, increasing BE values from 36.5% to 63.6%.SF 3 followed SF 4 and 6 in terms of FW/bag, but with law significance in % of DW and high significant results in stipe parameters which mean low quality as mentioned by Chukwurah et al.(2013), where the stipe length is considered a crucial quality indicator; mushrooms with shorter, wider stipe are generally preferred for their enhanced shelf life and marketability. From the aforementioned results, it can be deduced that SF8, followed by SF7 and SF10 (all chia-based formulation), represented the best-performing treatments in this study. These formulations revealed high quality and productivity, with BE values (36.63–43.27%) and FW per bag (85.15–137.57 g) falling within or exceeding the ranges reported for high-yield *Pleurotus* cultivation (30–100% BE and 70–150 g FW/bag) in previous studies (Pathmashini et al. 2009; Royse et al. 2017). Similar reference values for pileus width, stipe dimensions, and proximate composition have been reported by Miles and Chang (2004), further supporting the superior performance of these chia-based substrates. This suggests that the high FW/bag observed in SF3 was primarily driven by the elongation of the stipe. However, longer stipes is generally less preferred in commercial markets due to a lower cap: stipe ratio, as wider pilei with shorter stipes are favored by consumers (Pathmashini et al. 2009; Manzi et al. 2001). Based on the overall results, biological efficiency (BE) was considered the primary endpoint, indicating that SF8, followed by SF7 and SF10, were the most favorable formulations in terms of both productivity and acceptable quality traits.

Based on these findings, SF 8 demonstrated the most favorable nutritional profile, particularly in terms of protein content. Energy values were estimated considering the contents of crude protein, carbohydrates (each providing 4 kcal per gram), and fat (providing 9 kcal per gram). The energy content across samples ranged from 250.31 to 359.65 kcal per 100 g.

This can be explained by the fact that excessively high water-holding capacity (WHC) reduces aeration and limits oxygen diffusion inside the substrate. When pore spaces are saturated with water, anaerobic conditions may develop, which suppress mycelial respiration and slow down enzymatic degradation of lignocellulosic material, leading to lower yields (*P* > 0.05). Crop residues such as corn stalks, wheat straw and sunflower stalks are examples of materials with high WHC due to their fibrous structure, high cellulose and hemicellulose content, and slower degradation rates. While moderate WHC is beneficial to maintain substrate moisture, excessively high WHC results in compaction and poor gas exchange, which negatively affects mushroom productivity. Therefore, combining materials with high WHC and those with lower WHC (e.g., rice straw) is necessary to achieve a balanced substrate formulation.

### Chemical analysis of mushroom powder

Protein is one of the most important nutrients in mushrooms, and its content varied significantly among different substrate formulations. The inclusion of chia residues enhanced protein content. This enhancement can be explained by the nutritional profile of chia residues, which provide relatively higher crude protein and balanced carbon-to-nitrogen sources compared with conventional substrates such as rice straw. These characteristics stimulate fungal metabolism and promote protein accumulation in the fruiting bodies. A nutritional profile characterized by lower carbohydrate levels (*P* > 0.05), combined with higher protein, fiber, and ash content, and indicates a nutrient-dense profile (Maćkowiak et al. 2016; Ayimbila and Keawsompong 2023). However, implications for health require consideration of dietary context and further studies on bioavailability and clinical outcomes. Results revealed in Table 4 indicated that SF8 gave the highest percentage of protein and ash, while SF8, SF9, and SF10, which all contain chia residues, gave the highest percentages of fibers and the lowest percentage of carbohydrate (*P* > 0.05) when compared with the control treatment SF1 and conventional cultivation formulations SF4 and SF5. The results agreed with the findings of Alexei et al. (2022), who proved that chia residues are rich in dietary fiber, proteins, and essential fatty acids, which were used to estimate the biomass quality of chia residues (including stalks, leaves, and seed remnants) as a cellulosic material. They reported that it contains 107 g/kg crude protein, 80 g/kg ash, and 347 g/kg crude fat. These values refer to the whole-plant residues after seed harvesting, rather than stalks alone, and were used here to characterize the nutritional composition of CR: 348 g/kg acid detergent fiber, 517 g/kg neutral detergent fiber. This promotes the advantages of chia residues as a substrate for mushroom cultivation. Kumari and Atri (2014) reported an energy value of 364.7 kcal/100 g for oyster mushroom, which exceeds the values observed in this study while being comparable with other wild edible mushrooms. These results are consistent with previous research by Bano and Rajarathnam (1988); Dikeman et al. (2005); Abou Raya et al. (2014); Yasmin et al. (2015) and Oluwafemi et al. (2016).

Phenolic compounds, flavonoids, and antioxidants are recognized as important non-essential dietary phytochemicals that have been associated with various health benefits, including antioxidant activity, anti-inflammatory effects, and potential roles in reducing the risk of chronic diseases such as cardiovascular disorders and diabetes (Afiukwa et al. 2013; Upadhyaya et al. 2017). Giang et al. (2025) found that Phenolic compounds, flavonoids were 5.74 g TAE/100 g, 0.73 g QE/100 g of Oyster mushroom (*Pleurotus sajor-caju*) respectively Harun et al. (2025) used microwave-assisted extraction method for measuring phenolic compound profiling in diverse oyster mushrooms (*Pleurotus* spp.) they identified many compounds including one detected flavonoid. Gil-Ramírez et al. (2016) mentioned that “Up to October 2015, more than 136 reports ensured that mushrooms contain flavonoids”, while, On the other hand, they assumed that mushrooms do not possess the biosynthetic pathway for authentic flavonoid synthesis. If that correct, then the detected values likely correspond to compounds with flavonoid-like reactivity in the assay, rather than true flavonoids.

Within this context, SF8 and SF10 (both containing 64% chia residues) and SF9 (containing 64% corn stalks) demonstrated the most potent sources of bioactive compounds, indicating that substrate composition strongly influences the phenolic-related antioxidant potential of the fruiting bodies.

The inclusion of chia residues in the substrates positively affected all studied parameters. Mycelial growth and yield were significantly higher in SF8, SF9, and SF10 compared to conventional substrates. Total phenolic and flavonoid contents, as well as antioxidant activity, increased notably, exceeding typical values reported for mushrooms grown on standard substrates (phenolics 5–15 mg GAE/g; flavonoids 2–8 mg QE/g). Mineral analysis revealed elevated levels of microelements, indicating enhanced nutritional quality. These findings are consistent with studies using other plant-based substrates conducted by Mkhize et al. (2022) highlighting the biological significance and potential practical applications of chia residues in mushroom cultivation.

No recent studies demonstrated that mushrooms cultivated on substrates enriched with chia residues exhibited significantly higher antioxidant activity or mineral content compared to those grown on conventional substrates. The study of Jiménez et al. (2018) provides insight into the high percentages of these valuable components in chia residues, where they examined chia residues (waste containing remnants of leaves, stems, and some seeds) as rabbit fodder. They found that chia residues positively influenced the rabbits’ immune system, with anti-inflammatory Interleukin-10 (IL-10) and Tumor Necrosis Factor (TNF-α) gene expression increasing 22.4-fold when chia residue inclusion levels reached 40%. Further studies are needed to investigate the bioactive components in chia residues and their influence not only on different types of mushrooms but also on animal health. In the same context, the mineral content of mushroom powder, as shown in Table 5, reveals that chia residue-based substrates, such as SF8, SF9, and SF10, yielded the highest percentages of most measured elements, particularly microelements, indicating that adding chia residues enhances the nutritional value of the formulated substrates. More studies should be conducted to explore the bioactive components of chia residues and their impact on the nutritional quality, antioxidant activity, and mineral composition of mushrooms, as well as potential benefits for animal health.

Beyond similarities with previous reports, the observed differences among substrate formulations can be mechanistically explained by the physicochemical properties of chia residues. Their relatively high protein, fiber, and ash content, along with a balanced carbon-to-nitrogen ratio, favor fungal metabolism and enhance the nutritional value of the fruiting bodies. In contrast, substrates with excessively high water-holding capacity reduce aeration and oxygen availability, which limits mycelial respiration and results in lower yields. Likewise, variation in primordium initiation among formulas is linked to the accessibility of cellulose and hemicellulose in chia residues, which are more readily degraded than lignin-rich components of conventional substrates. These interpretations provide a deeper understanding of the reasons behind the observed phenomena and highlight the value of chia residues as a sustainable substrate for mushroom cultivation. In addition to the nutritional and bioactive improvements observed, the use of chia residues in mushroom cultivation has important environmental implications. Integrating chia residues with other organic materials can help farmers develop more environmentally friendly and productive cultivation practices. This strategy optimizes resource utilization, reduces agricultural waste, and aligns with sustainable agriculture principles. By valorizing plant residues as a substrate component, the approach contributes to circular agriculture and minimizes environmental impacts associated with conventional waste disposal.

## Conclusion and recommendations

Our results suggest that chia plant residues can serve as effective components in substrate formulations for cultivating *Pleurotus ostreatus* var. florida under greenhouse conditions. The results of this study indicate that mixtures of chia residues and rice straw, particularly at intermediate ratios, supported higher productivity of *Pleurotus ostreatus* var. florida 14. Among the tested formulations, SF8 (64% RS + 22% CR) achieved the highest biological efficiency and favorable compositional attributes, followed by SF7 and SF10. SF9 (64% corn stalks) and SF10 (64% chia residues) also showed high levels of bioactive components, ranking just below SF8. To our knowledge, this is the first report examining chia residues as substrates for *Pleurotus ostreatus* cultivation and their effects on productivity, quality, bioactive components, antioxidant activity, and mineral content.

More studies are needed to investigating chia residues-based formulation for cultivating different species of oyster mushroom or other types of mushrooms. Cultivating mushrooms on diverse substrates such as chia residues can further augment their nutritional value, potentially elevating antioxidant activity and phenolic compound content. Incorporating chia residues into mushroom cultivation substrates may represent a sustainable valorization pathway for agricultural by-products, though pilot-scale and on-farm trials are required to validate economic feasibility. This integrated approach allows farmers to combine chia residues with other organic materials, promoting environmentally friendly and productive cultivation practices, optimizing resource utilization, and minimizing waste in line with sustainable agriculture principles.

## Data Availability

No datasets were generated or analysed during the current study.

## Abbreviations

CR: Chia Residues
CC: Corn Cobs
GP: Gypsum Powder
BE: Biological Efficiency
FW: Fresh Weight
K: Potassium
P: Phosphorus
Na: Sodium
RS: Rice Straw
SFs: Substrate formulations
GAE: Gallic Acid Equivalent
DPPH: 2, 2-Diphenyl-1-picrylhydrazyl
DW: Dry Weight
Mg: Magnesium
Fe: Iron
Se: Selenium
